# Portable Force Plates: A Viable and Practical Alternative to Rapidly and Accurately Monitor Elite Sprint Performance

**DOI:** 10.3390/sports6030061

**Published:** 2018-07-12

**Authors:** Irineu Loturco, Lucas A. Pereira, Ronaldo Kobal, Cesar C. Cal Abad, Victor Fernandes, Rodrigo Ramirez-Campillo, Timothy Suchomel

**Affiliations:** 1 Nucleus of High Performance in Sport–NAR, Sao Paulo 04753060, Brazil; lucasa_pereira@outlook.com (L.A.P.); rokorin2002@hotmail.com (R.K.); c.cavinato@uol.com.br (C.C.C.A.); 2 B3 Track & Field Club, Sao Paulo 04753060, Brazil; victor.fernandescoach@bol.com.br; 3 Association of High-Performance Training & Sports Development–ADAPT, Sao Paulo 04753060, Brazil; 4 Research Nucleus in Health, Physical Activity and Sport, Department of Physical Activity Sciences, Universidad of Los Lagos, Osorno 5290000, Chile; r.ramirez@ulagos.cl; 5 Department of Human Movement Sciences, Carroll University, Waukesha, WI 53186, USA; timothy.suchomel@gmail.com

**Keywords:** force platform, kinetic measures, speed, muscle power, rate of force development, strength, plyometric, stretch-shortening cycle

## Abstract

This study aimed to examine the associations between a series of mechanical variables automatically generated by a portable force plate (PFP) and the actual performance of professional sprinters over a 150 m course. To test these correlations, 12 top-level sprinters performed vertical jumps (squat and countermovement jumps; SJ and CMJ, respectively), a 60 m sprint test, and a 150 m sprint test. Pearson product-moment coefficient of correlation and multiple regression analyses were used to determine the relationships between the sprinting velocities and vertical jump outputs. The SJ parameters were moderately to near perfectly associated with the different sprint distances, and the SJ height presented the highest correlation scores (*r* = 0.90 with velocities over 10- and 20-m). The correlation coefficients between the CMJ outcomes and sprint results varied between moderate and very large (from 0.38 to 0.88). Finally, the coefficients of determination (R^2^) ranged from 0.71 to 0.85 for the different multiple regressions involving PFP automatic measures. The PFP can provide practitioners with quick and accurate information regarding competitive athletes. Due to the strong correlations observed, coaches are encouraged to frequently adjust and tailor the training strategies of their sprinters, using practical and timesaving PFP measurements.

## 1. Introduction

The actual performance of professional sprinters is one of the most studied issues in sport science [1,2,3,4]. In part, this may be explained by two different factors: (1) the key contribution of speed ability to specific performance in many sport disciplines [5,6,7,8,9]; and (2) the inherent difficulties of enhancing maximum sprint capacity in elite sprinters (e.g., Olympians), who usually depend on hundredths of a second to achieve positive outcomes during competitions [10]. Therefore, a better and more comprehensive understanding of this important physical trait may benefit not only track and field experts, but also coaches from several sports, who need to improve the competitive level of their athletes. 

In recent years, a number of investigations involving world-class sprinters have been conducted in an attempt to extract more consistent information about their exceptional performances [2,4,11]. Being aware of the typical limitations associated with the experimental conditions in studies performed at the top-level, sport scientists have been successfully employing cross-sectional designs for such purposes. For example, it was reported that the orientation of the resultant ground reaction force over the acceleration phase of a sprint is more important to elite sprinters’ performance than its total amount [3]. Moreover, it seems that the simple measurement of vertical jump height or horizontal jump distance may provide coaches with relevant data regarding the actual performance obtained by professional sprinters in 100 m dash events [12]. Based on these results, collected with contact mats or instrumented treadmills, practitioners can effectively adjust and control the training programs of their athletes, focusing on their individual needs and performance variations.

Another practical way to assess track and field athletes is through the use of standard force plates which acquire forces and moments in three directions (i.e., *x*, *y*, and *z* axis), providing coaches with additional information (i.e., kinetic and kinematic data) related to common field tests (e.g., vertical jumps) [13]. For instance, Coh and Mackala [14] revealed that elite sprinters could jump higher, reach higher take-off velocity, and produce greater concentric impulse during countermovement jump (CMJ) measurements than their subelite peers. Similarly, the kinetic variables underpinning drop jump performance were able to discriminate between highly-trained sprint athletes and nonsprint-trained subjects, in a study that examined a substantial number of outputs gathered during the braking and propulsive movement phases [1]. Although very relevant and useful, the complex evidence derived from these works along with the complicated procedures usually related to force place data acquisition (and extraction) might hamper the use of such information as an input for decision making in athletic training settings [15].

The use of portable force plates (PFP)—which are capable of automatically and accurately detecting mechanical parameters via custom-designed software [16]—is a possible way to reduce the complexity of these laborious and time-consuming methodological processes. In a previous study with top-level sprinters and PFP, Loturco et al. [8] reported that jump distances and peak forces (PF) in vertical and horizontal jumps are significantly related to sprinting speed. Nevertheless, the correlational analyses were performed without considering other critical and “immediate” mechanical outputs (e.g., rate of force development (RFD) and peak power (PP)) as independent variables. Furthermore, the authors tested the athletes over a 60 m course, which could compromise the extrapolation of these findings to greater distances (e.g., 150 m). Thus, the aim of this study was to examine the relationships between a series of mechanical variables automatically generated by a PFP (PF, RFD, PP, and jump height) and the actual performance of professional sprinters over a 150-m course. Based on mechanistic principles, we hypothesized that higher PFP outputs would be closely associated with elite sprint performance.

## 2. Materials and Methods

### 2.1. Testing Procedures

Athletes executed the tests on the same day, in the following order: vertical jumps comprising squat jump (SJ) and CMJ; a 60-m sprint; and a 150-m sprint test. Subjects were instructed to maintain their nutritional and sleep habits and arrive at the sports laboratory in a fasting state for at least 2-h, avoiding alcohol and caffeine consumption for at least 48-h before the tests. Due to their constant assessments in our facilities, all subjects were previously familiarized with the testing procedures. A standardized warm-up comprising light to moderate self-selected runs for 5-min was performed before the tests. Sub-maximal attempts at each test were also executed prior to the maximal tests. Between each test, a 15-min resting interval was implemented to explain the next measurements and adjust the testing devices. Participants performed low intensity activities during the rest interval to maintain readiness for the next test. All physical tests were performed between 8:00 a.m. and 12:00 p.m.

### 2.2. Subjects

Twelve top-level sprinters (7 men: age: 22.9 ± 5.3 years; height: 181.6 ± 9.6 cm; body mass: 76.3 ± 10.3 kg; and 5 women: age: 21.5 ± 3.1 years; height: 164.3 ± 10.1 cm; body mass: 55.4 ± 3.7 kg) participated in this study. The sample comprised athletes who have been involved in Olympic Games, World Championships, Pan-American, and South-American competitions, attesting their high level of performance and competitiveness. Prior to participating in this study, athletes were briefed on the experimental design and signed an informed consent form. This study was performed in accordance with the ethical standards of the Helsinki Declaration and was approved by the local Ethics Committee.

### 2.3. Vertical Jumps

**Figure 1:**
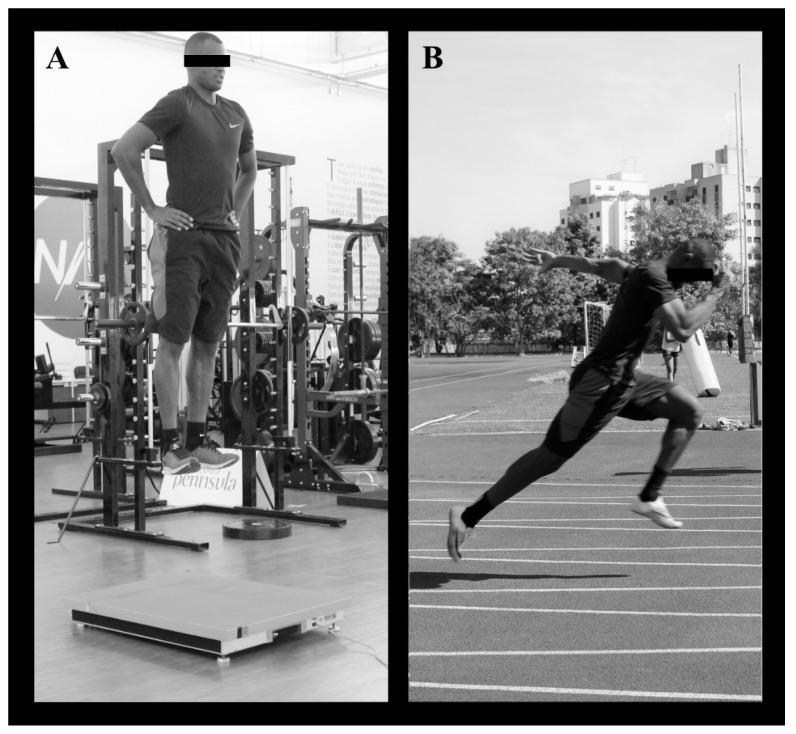
An Olympic sprinter with a best performance of 10.11 s in the 100 m dash at two different moments: (**A**) jumping 64 cm in a vertical jump test on a force plate; and (**B**) during the initial acceleration phase of the 60 m sprint test.

Vertical jumps were assessed using SJ and CMJ. In the SJ, athletes were required to achieve a squat position with 90° of knee flexion and hold this position for ~2 s before jumping, without any preparatory movement. In the CMJ, athletes were instructed to execute a downward movement followed by complete extension of the hip, knee, and ankle joints, and were free to determine the countermovement amplitude to avoid changes in jumping coordination. All jumps were executed with the hands on the hips and the athletes were instructed to jump as high as possible. A total of five attempts were allowed for each jump, interspersed by 15-s intervals. The jumps were performed on a PFP (AccuPower, AMTI, Watertown, MA, USA), which sampled at a rate of 400 Hz (Figure 1A). The following mechanical variables were automatically obtained by the custom-designed software of the PFP: jump height; PF; PP; time to PF; and RFD between 0–50 ms (RFD_0–50_), 0–100 ms (RFD_0–50_), and 0 ms to the PF (RFD_0–PF_). The best attempt at each jump was used for the analyses.

### 2.4. Sprinting Velocity

For the 60 m sprint test, five pairs of photocells (Smart Speed, Fusion Equipment, Brisbane, Australia) were positioned at distances of 0, 10, 20, 40, and 60 m along the sprinting course (Figure 1B). Meanwhile, for the 150 m sprint test, three pairs of photocells were positioned at distances of zero, 100 m, and 150 m along the sprinting course. Athletes performed two 60 m sprints and two 150 m sprints starting from a standing position 0.3 m behind the starting line. The sprint tests were performed on an official running track. An 8-min rest interval was allowed between the two attempts and the fastest time was considered for the analyses.

### 2.5. Statistical Analyses

Data are presented as means ± standard deviation. The normality of data was tested using the Shapiro-Wilk test. For the comparisons between male and female athletes, a number of independent *t*-tests were applied separately for each performance variable. Pearson product-moment coefficient of correlation was used to determine the relationships between the performances in the sprinting velocities and the vertical jump outcomes. The threshold used to qualitatively assess the correlations was based on the following criteria: <0.1, trivial; 0.1–0.3, small; 0.3–0.5, moderate; 0.5–0.7, large; 0.7–0.9, very large; >0.9 nearly perfect [17]. A multiple regression analysis was performed to predict the sprint velocities, using as independent variables the SJ height and RFD. These variables were selected because they did not violate the collinearity diagnosis (variance inflation factor < 10 and tolerance > 0.2) [18,19,20] and presented the best combination among all variables tested to predict the sprint velocities. The level of significance was set at *p* < 0.05. The analyses were performed using IBM SPSS Statistics for Windows, Version 20.0 (IBM Corp., Armonk, NY, USA). All performance tests presented good levels of absolute and relative reliability (CV < 5% and ICC > 0.90 for all assessments) [17].

## 3. Results

All data presented normal distribution. The times to reach PF during both vertical jumps were not significantly different when comparing male and female athletes (228.6 ± 39.3 ms vs. 217.0 ± 32.3 ms in the SJ; and 214.2 ± 26.5 ms vs. 228.8 ± 24.3 ms in the CMJ; for male and female athletes, respectively); *p* > 0.05. Table 1 demonstrates the comparisons of the variables obtained from the PFP in the vertical jumps between male and female athletes. Male athletes demonstrated higher scores in all variables when compared to female sprinters (*p* < 0.05), with the exception of RFD_0–50_ for the CMJ (*p* > 0.05). Table 2 shows the comparisons of the sprinting velocity (VEL) achieved in the different distances. Male athletes demonstrated higher sprint velocities in all distances tested than female athletes (*p* < 0.05).

Table 3 presents the correlation coefficients between sprint velocities and PFP-derived variables from vertical jumps. The SJ outcomes were moderately to near perfectly associated with the different sprint distances. The SJ height presented the highest correlation scores (*r* = 0.90 between SJ height and VEL 10 and 20 m). The correlation coefficients between the CMJ outcomes and the different sprint distances varied between moderate and very large.

Table 4 demonstrates the multiple regression analysis combining SJ height and RFD as independent variables to predict the sprint velocities. The coefficient of determination varied between 0.71 and 0.83, 0.80 and 0.83, and 0.81, and 0.85, for the combination between SJ height and RFD_0–50_, RFD_0–100_, and RFD_0–PF_, respectively (*p* < 0.01 for all multiple regression analysis).

## 4. Discussion

This study examined the relationships between certain PFP outputs (i.e., PF, PP, RFD, and jump height) and the actual performance of professional sprinters over a 150 m course. Overall, these mechanical parameters were closely associated with the maximum running speed measured at different distances, with slight variations among them (Table 3). Of note, when combined in multiple regression models, some variables accounted for 80 to 85% (i.e., SJ height + RFD_0–100_ and SJ height + RFD_0–PF_) of the variance for all dependent variables (i.e., sprinting speed at 10, 20, 40, 60, 100, and 150 m) (Table 4). These “nearly perfect correlations” (*r* ≥ 0.90) [17] between kinetic and kinematic measures are possibly the strongest observed in investigations performed with elite sprinters across this comprehensive range of distances.

PF, PP, and jump height (collected by force plates in SJ and CMJ attempts) have been shown to be significantly related to the performance obtained by top-level sprinters in maximal sprint tests [8,21,22]. Our data corroborate these findings, emphasizing that the ability to apply force (and produce power) against the ground is critical to achieve greater speeds while sprinting [6,23]. The same holds true for the vertical jump height; a measure able to express values “already normalized” to the subjects’ body mass [12]. Theoretically, if during a vertical jump an athlete jumps higher, he produces higher values of relative force and power (N·kg^−1^ and W·kg^−1^) than his weaker counterpart. That said, it is plausible to assume that these mechanical outputs are associated with the sprinters’ performance, since they have to accelerate their bodies forward as quickly as possible during training and competitions, applying great amounts of force onto the ground. Curiously, the associations between PF (in both SJ and CMJ tests) and speed described here are greater than those reported in a previous study involving PFP and top-level sprinters [8]. Although it is difficult to explain such discrepancies, it is essential to mention that: (1) our subjects trained under the supervision of the same technical and scientific staff and were performing an identical plyometric training program at the data collection phase, and (2) the present investigation was conducted during the in-season competitive period (contrary to the latter, where athletes were tested “at the beginning of the preseason”) [8]. Therefore, it is possible to suppose that both training status and strategies may critically affect the relationships between different mechanical parameters related to elite sprint performance.

The RFD is defined as the slope of the force-time curve (Δforce/Δtime) obtained during a given movement and has been described as an important factor to achieve maximum performance in a range of power-based sports (e.g., sprinting events) [24,25]. In this study, we used three different RFD slopes (i.e., 0–50 ms, 0–100 ms, and 0–PF) to evaluate the athletes, and the longer intervals (i.e., RFD_0–100_ and RFD_0–PF_) have been shown to be more associated with performance over the entire 150-m course (Table 3), including acceleration, top-speed, and deceleration phases. To some extent, this can be explained by analyzing the variations in foot contact times with the ground among the different phases of sprinting, which typically range from 80 to 200 ms (for maximum velocity and initial acceleration, respectively) [26,27]. Thereby, although very limited, the time to apply force throughout a sprint race is always above 50 ms, highlighting the necessity of using longer RFD intervals to properly assess the force-time characteristics of elite sprinters.

To our knowledge, this is the first investigation to use certain mechanical variables automatically generated by a PFP to predict sprint performance through the use of different linear regression analyses across a course of 150 m. Even considering the slight differences among the simple and multiple correlations presented here (Table 3 and Table 4), it is crucial to ponder that our sample is composed of top-level sprinters and, for this selected group, minimal differences in performance are of critical importance to achieve great results [28]. The combination of a variable related to force-time parameters (i.e., RFD) [24,29] with a measure already adjusted for the body mass (i.e., jump height) [12] in the same predictive model could provide practitioners with more comprehensive and balanced information regarding elite sprinters’ performance. From a practical perspective, this allows track and field coaches to easily and simultaneously examine the athletes’ ability to rapidly produce high levels of relative force and power, which is recognized to be essential for reaching high sprinting velocities over short time intervals [2,6,24].

This study is limited by its cross-sectional nature, small-sample size, and the particular characteristics of the sample (i.e., professional sprinters), which may hamper the extrapolation of our data to less specialized populations. As an illustration, during the speed tests, the average time for male sprinters was (impressive) 10.75 s at the 100 m distance. Despite these inherent particularities and limitations, we believe that our findings can help sport scientists to create better testing and training strategies for this group of elite athletes who continuously train and compete at the upper limits of human performance.

## 5. Conclusions

The use of standard force plates to assess athletic performance is common and widespread in sport science. Nevertheless, the process of data acquisition, extraction and analysis is not straightforward, which could compromise their use in applied sport settings. Alternatively, the PFP can provide practitioners with quick, useful, and accurate information regarding competitive athletes, namely professional sprinters. With this in mind, sport scientists may use the data automatically generated by this device to evaluate and predict sprint running performance, by using its mechanical outputs in simple or multiple linear regression models. According to our results, these measures can account for up to 85% (i.e., SJ height + RFD_0–PF_) of the variance for the maximal sprinting speed assessed at different distances, from 10 to 150 m. Due to these strong correlations, track and field coaches are encouraged to frequently adjust and tailor the training strategies of their top-level sprinters, based on practical and timesaving PFP measurements. Further studies should be conducted to examine the causal relationships between the meaningful variations in PFP outputs and actual sprint performance.

## Figures and Tables

**Table 1:** Comparisons of the variables obtained from the portable force plate in the vertical jumps between male and female athletes.

	SJ		CMJ	
	Male	Female	Male	Female
Height (cm)	50.5 ± 3.6 *	39.9 ± 2.8	51.7 ± 4.9 *	40.8 ± 2.0
Peak Force (N)	2084.0 ± 302.0 *	1410.0 ± 135.1	2195.0 ± 258.1 *	1342.0 ± 127.7
Peak Power (W)	5169.6 ± 731.1 *	3087.6 ± 262.1	4899.4 ± 747.8 *	2891.0 ± 131.4
RFD_0–50_ (N·s^−1^)	6788.6 ± 1359.4 *	4784.0 ± 1687.0	4960.0 ± 1109.4	3384.0 ± 2085.5
RFD_0–100_ (N·s^−1^)	8771.4 ± 1544.5 *	5842.0 ± 1672.0	8081.4 ± 1939.6 *	5102.0 ± 3243.5
RFD_0–PF_ (N·s^−1^)	6662.2 ± 1558.5 *	4397.0 ± 1474.8	9731.4 ± 841.0 *	5675.3 ± 1088.1

Note: SJ: squat jump; CMJ: countermovement jump; RFD: rate of force development; PF: peak force. * *p* < 0.05 comparing male and female athletes.

**Table 2:** Comparisons of the sprinting velocity (VEL) achieved in the different distances between male and female athletes.

	Male *	Female
VEL 10-m (m·s^−1^)	6.08 ± 0.15	5.50 ± 0.27
VEL 20-m (m·s^−1^)	7.20 ± 0.15	6.49 ± 0.16
VEL 40-m (m·s^−1^)	8.30 ± 0.21	7.37 ± 0.06
VEL 60-m (m·s^−1^)	8.87 ± 0.27	7.77 ± 0.03
VEL 100-m (m·s^−1^)	9.30 ± 0.28	8.01 ± 0.15
VEL 150-m (m·s^−1^)	9.40 ± 0.36	7.98 ± 0.22

* *p* < 0.05 for all comparisons between male and female athletes.

**Table 3:** Correlation coefficients between sprint velocities and portable force platform derived variables from vertical jumps.

		VEL 10 m	VEL 20 m	VEL 40 m	VEL 60 m	VEL 100 m	VEL 150 m
SJ	height	0.90 *	0.90 *	0.88 *	0.85 *	0.83 *	0.80 *
	PF	0.68 *	0.78 *	0.81 *	0.83 *	0.83 *	0.85 *
	PP	0.80 *	0.85 *	0.86 *	0.86 *	0.86 *	0.86 *
	RFD_0–50_	0.46	0.53	0.56	0.58 *	0.64 *	0.67 *
	RFD_0–100_	0.53	0.65 *	0.71 *	0.74 *	0.79 *	0.82 *
	RFD_0–PF_	0.62 *	0.75 *	0.81 *	0.84 *	0.84 *	0.89 *
CMJ	height	0.86 *	0.88 *	0.86 *	0.84 *	0.83 *	0.80 *
	PF	0.80 *	0.83 *	0.83 *	0.83 *	0.84 *	0.83 *
	PP	0.80 *	0.84 *	0.84 *	0.85 *	0.85 *	0.86 *
	RFD_0–50_	0.40	0.46	0.44	0.44	0.38	0.39
	RFD_0–100_	0.61 *	0.57	0.51	0.48	0.40	0.38
	RFD_0–PF_	0.76 *	0.77 *	0.76 *	0.75 *	0.74 *	0.71 *

Note: VEL: velocity; SJ: squat jump; CMJ: countermovement jump; PF: peak force; PP: peak power; RFD: rate of force development; * *p* < 0.05.

**Table 4:** Multiple regression analysis combining squat jump (SJ) height and rate of force development (RFD) as independent variables to predict (R^2^) sprinting velocity in elite sprinters.

	SJ Height + RFD_0–50_				SJ Height + RFD_0–100_				SJ Height + RFD_0–PF_			
	*B*	*SE*	R^2^	SEE	*B*	*SE*	R^2^	SEE	*B*	*SE*	R^2^	SEE
VEL 10-m	3.26	0.43	0.83	0.16	3.20	0.44	0.83	0.16	3.22	0.47	0.82	0.16
VEL 20-m	4.32	0.41	0.82	0.18	4.36	0.40	0.83	0.18	4.46	0.42	0.83	0.18
VEL 40-m	4.70	0.55	0.80	0.25	4.79	0.51	0.83	0.23	5.03	0.52	0.84	0.22
VEL 60-m	4.70	0.72	0.76	0.32	4.84	0.65	0.81	0.29	5.20	0.64	0.83	0.27
VEL 100-m	4.51	0.97	0.74	0.41	4.73	0.84	0.81	0.35	5.07	0.87	0.81	0.34
VEL 150-m	4.50	1.21	0.71	0.49	4.81	1.01	0.80	0.40	5.35	0.93	0.85	0.35

Note: VEL: velocity; *B*: beta estimate; *SE*: standard error; SEE: standard error of estimate; *p* < 0.01 for all multiple regression analysis.
